# Biochemical and functional characterization of a recombinant monomeric factor VIII–Fc fusion protein

**DOI:** 10.1111/jth.12076

**Published:** 2013-01

**Authors:** R T PETERS, G TOBY, Q LU, T LIU, J D KULMAN, S C LOW, A J BITONTI, G F PIERCE

**Affiliations:** Biogen Idec HemophiliaWaltham, MA, USA

**Keywords:** Factor VIII, Fc fusion, hemophilia A, long-acting, rFVIIIFc

## Abstract

*Background:* Hemophilia A results from a deficiency in factor VIII activity. Current treatment regimens require frequent dosing, owing to the short half-life of FVIII. A recombinant FVIII–Fc fusion protein (rFVIIIFc) was molecularly engineered to increase the half-life of FVIII, by 1.5–2-fold, in several preclinical animal models and humans. *Objective:* To perform a biochemical and functional in vitro characterization of rFVIIIFc, with existing FVIII products as comparators.*Methods:* rFVIIIFc was examined by utilizing a series of structural and analytic assays, including mass spectrometry following lysyl endopeptidase or thrombin digestion. rFVIIIFc activity was determined in both one-stage clotting (activated partial thromboplastin time) and chromogenic activity assays, in the context of the FXase complex with purified components, and in both in vitro and ex vivo rotational thromboelastometry (ROTEM) assays performed in whole blood. *Results:* rFVIIIFc contained the predicted primary structure and post-translational modifications, with an FVIII moiety that was similar to other recombinant FVIII products. The von Willebrand factor-binding and specific activity of rFVIIIFc were also found to be similar to those of other recombinant FVIII molecules. Both chromogenic and one-stage assays of rFVIIIFc gave similar results. Ex vivo ROTEM studies demonstrated that circulating rFVIIIFc activity was prolonged in mice with hemophilia A in comparison with B-domain-deleted or full-length FVIII. Clot parameters at early time points were similar to those for FVIII, whereas rFVIIIFc showed prolonged improvement of clot formation. *Conclusions:* rFVIIIFc maintains normal FVIII interactions with other proteins necessary for its activity, with prolonged in vivo activity, owing to fusion with the Fc region of IgG_1_.

## Introduction

Hemophilia A is an X-chromosome-linked bleeding disorder characterized by a deficiency of functional clotting factor VIII [1]. Individuals with hemophilia A experience prolonged bleeding after trauma, and recurrent spontaneous bleeds into the soft tissue and joints, leading to joint damage and severe disability. Hemophilia A is treated with replacement therapy with either plasma-derived FVIII or recombinant FVIII (rFVIII) products. The current prophylaxis standard of care utilizes repeated treatments with the replacement factor, typically three times per week or every other day, to maintain FVIII levels at ≥ 1%. A long-lasting FVIII molecule that can maintain prophylaxis with reduced frequency of injections, and potentially reduce the number of infusions needed to control a bleed on demand, may be useful to improve treatment outcomes.

FVIII is synthesized as an approximately 300-kDa (2332 amino acids) single-chain (SC) protein that consists of the structural domains A1–A2–B–A3–C1–C2 [2], including a large B domain with no known function or homology to other proteins. The B domain is normally processed intracellularly at various positions to generate a heavy chain (HC) (A1–A2–B) varying from 90 to 200 kDa in size and an 80-kDa light chain (LC) (A3–C1–C2) that remain associated via metal ion-mediated, non-covalent interactions [3]. Deletion of a large portion of the B domain from Ser743 to Gln1638 has no effect on the functional activity of FVIII, but decreases the size of the protein significantly (38% reduction) to a 90-kDa HC and an 80-kDa LC, and increases FVIII expression levels in eukaryotic cells [4,5].

We have generated a novel recombinant fusion protein comprising a single molecule of B-domain-deleted FVIII (BDD FVIII) fused to the dimeric human Fc region from IgG_1_ to extend the half-life of FVIII. The Fc domain enables the fusion protein to bind to the neonatal Fc receptor (FcRn) [6], and thus leverages the same natural pathway that is responsible for the long plasma half-life of immunoglobulin molecules by protecting the protein from intracellular degradation. We have shown previously that the monomeric Fc fusion protein extends the half-life of FVIII in mouse and dog models of severe hemophilia A by approximately two-fold, as well as in humans by 1.5–1.7-fold [6,7]. Herein, we describe the biochemical and functional characterization of this rFVIII–Fc fusion protein (rFVIIIFc) molecule, including ex vivo data that confirm the prolonged half-life and clot integrity as compared with currently available FVIII products.

## Materials and methods

### Cloning, expression and purification of rFVIIIFc

**Figure 1:**
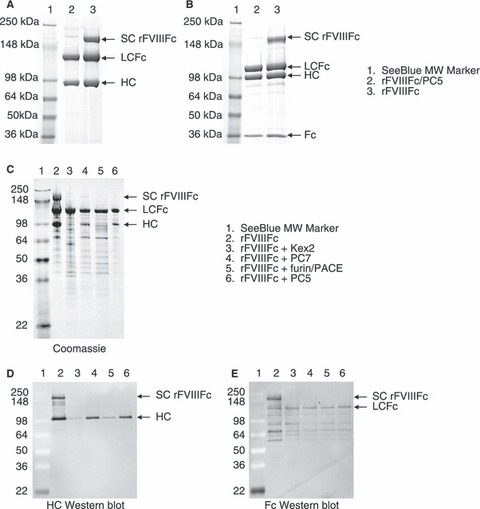
SDS-PAGE and Western blot analysis of recombinant factor VIII–Fc fusion protein (rFVIIIFc). rFVIIIFc was purified from cell lines stably transfected with rFVIIIFc alone or cotransfected with PC5. Protein was run on non-reducing (A) and reducing (B) SDS-PAGE gels, and visualized with Sypro Ruby staining. The heavy chain (HC), light chain (LC)Fc, single-chain (SC) rFVIIIFc and Fc bands are indicated. rFVIIIFc (synthesized as a single polypeptide with the two Fc regions connected by a Gly/Ser linker) was also produced in transient transfections, either alone or cotransfected with Kex2, PC7, extracellular domain of furin, or PC5; protein A pulldowns were analyzed by non-reducing SDS-PAGE followed by Coomassie staining (C) or western blotting for the FVIII HC (D) or IgG Fc region (E).

The expression plasmids for human BDD FVIII (Ser743 to Gln1638 fusion) fused to human IgG_1_ Fc with no intervening linker, and for the Fc chain alone, were generated by standard molecular biology techniques, and protein was purified from stably transfected HEK293 cells grown in serum-free suspension culture. For the transient transfection experiments in Fig. 1C–E, a second plasmid was constructed, expressing rFVIIIFc as a single polypeptide chain with dimeric Fc, in which the first Fc region is connected to the second Fc region with a Gly/Ser flexible linker, which produces only the rFVIIIFc monomer without free Fc.

### FVIII comparators

Three different sources of antihemophilic factor (recombinant) were utilized in these studies: recombinant BDD FVIII (rBDD FVIII) ReFacto and Xyntha (Wyeth Pharmaceuticals, Philadelphia, PA, USA) and full-length FVIII Advate (Baxter Healthcare Corporation, Westlake Village, CA, USA). Proteins were purchased and reconstituted according to the manufacturers’ guidelines; nominal values were used for activity dosing calculations, and verified by chromogenic activity assay.

### Thrombin and lysyl endopeptidase (LysC) peptide mapping

FVIII samples were fully digested with thrombin, reduced, and analyzed by either RP-HPLC–UV or RP-HPLC–mass spectrometry (MS). The peptide sequence was also confirmed with LysC peptide mapping and analyzed by RP-HPLC–MS.

### Activity assays

FVIII samples were analyzed with a one-stage clotting assay (Actin FSL reagent; Siemens, Dallas, TX, USA) or with a chromogenic assay (Siemens) on a Sysmex CA 1500 instrument, with multiple dilution analysis, and activity was calculated relative to rFVIIIFc reference standards that have been calibrated against the 7th or 8th International Standard for Human FVIII Concentrate.

### Activity in FXase complex

Methods for determining activity in FXase complex were as previously described [8], utilizing synthetic phospholipid vesicles (25% phosphatidylserine [PS]/75% phosphatidylcholine [PC]) or platelets (resting or activated with 50 μg mL^−1^ SFLLRN peptide/10 μm ADP), as indicated. Parameters for all assays are expressed as mean ± standard deviation, from three replicate runs each in duplicate, except for platelet assays performed in two replicate runs, each in duplicate. For activated protein C (APC) inactivation, thrombin-activated FVIII samples were treated with hirudin and then APC for 90 min before assaying of FXase activity. Normalized FXa generation rates were obtained from the mean of duplicate runs.

### Surface plasmon resonance (SPR) analysis of von Willebrand factor (VWF) binding

The affinities of rFVIIIFc and rBDD FVIII for human VWF were determined with a Biacore T100 instrument (GE Healthcare, Piscataway, NJ, USA) operated in single-cycle kinetic mode, as described in detail in the Materials and Methods section in the Supporting Information.

### Rotational thromboelastometry (ROTEM)

For in vitro ROTEM, rFVIII proteins were spiked in triplicate into citrated pooled blood collected from the vena cava of five to six male mice with hemophilia A [9] to final concentrations of 0%, 0.1%, 1%, 10% and 100% of the normal plasma FVIII level. The clot was initiated by the addition of CaCl_2_ (non-activated thromboelastometry [NATEM]), and clotting time (CT), clot formation time (CFT), α-angle and maximum clot firmness were recorded on the ROTEM system (Pentapharm, Munich, Germany).

For ex vivo ROTEM, male mice with hemophilia A were injected intravenously with a single dose of 50 IU kg^−1^ rFVIIIFc, Advate, or Xyntha, and five mice were killed at each time point (5 min, 24 h, 48 h, 72 h and 96 h post-dosing). Individual citrated whole blood collected from the vena cava was immediately analyzed by NATEM on the ROTEM system, and parameters were measured as above.

Statistical analysis was performed by two-way analysis of variance with Bonferroni post hoc tests in graphpad prism 5.0 (GraphPad, La Jolla, CA, USA). All animal studies were conducted in compliance with Institutional Animal Care and Use Committee-approved protocols.

Additional details of the methods are included in the Supporting Information.

## Results

### Expression and analysis of rFVIIIFc

rFVIIIFc was synthesized as two polypeptide chains, one chain consisting of BDD FVIII fused to the Fc domain of IgG_1_ and the other chain consisting of the same Fc region alone (Fig. S1). Although cells transfected with the rFVIIIFc/Fc dual expression plasmid were expected to secrete three products (rFVIIIFc dimer, rFVIIIFc monomer, and Fc dimer), only the rFVIIIFc monomer and Fc dimer were detected in conditioned medium, probably because of failure of dimerization and/or secretion of the approximately 400-kDa rFVIIIFc dimer. Purified rFVIIIFc was analyzed by non-reducing and reducing SDS-PAGE (Fig. 1A,B). For the non-reducing SDS-PAGE, bands were found migrating at approximately 90 and 130 kDa, which were consistent with the predicted molecular masses of the rFVIIIFc HC-dimeric and LC-dimeric Fc fusion (Fig. 1A, lane 3). A third band also was detected at approximately 220 kDa – this was consistent with the predicted molecular mass for SC FVIIIFc, in which the arginine at position 1648 is not cleaved during secretion. For the reduced SDS-PAGE analysis, major bands were seen migrating at approximately 25, 90, 105 and 195 kDa, which were consistent with the predicted molecular masses for the reduced Fc, HC, LCFc and SC FVIIIFc chains (Fig. 1B, lane 3). Cotransfection with human PC5, a member of the proprotein convertase subtilisin/kexin (PCSK)-type proteases, resulted in full processing and confirmed the identity of the SC FVIIIFc band (Fig. 1A,B, lane 2).

Transient transfection experiments demonstrated that cotransfection of rFVIIIFc with several different proprotein convertase family enzymes (e.g. human PC5 [PCSK5], human PC7 [PCSK7], the extracellular domain of human PACE/furin [PCSK3], or yeast homolog Kex2) resulted in full cleavage of the SC rFVIIIFc isoform, as demonstrated by non-reducing SDS-PAGE analysis (Fig. 1C). Kex2 and PACE/furin also appeared to degrade the HC to a significant extent, according to SDS-PAGE and HC and Fc western blotting analysis (lanes 3 and 5, Fig. 1C–E, respectively). Similar processing and evidence of degradation was found in transfection experiments with unconjugated FVIII (data not shown).

**Figure 2:**
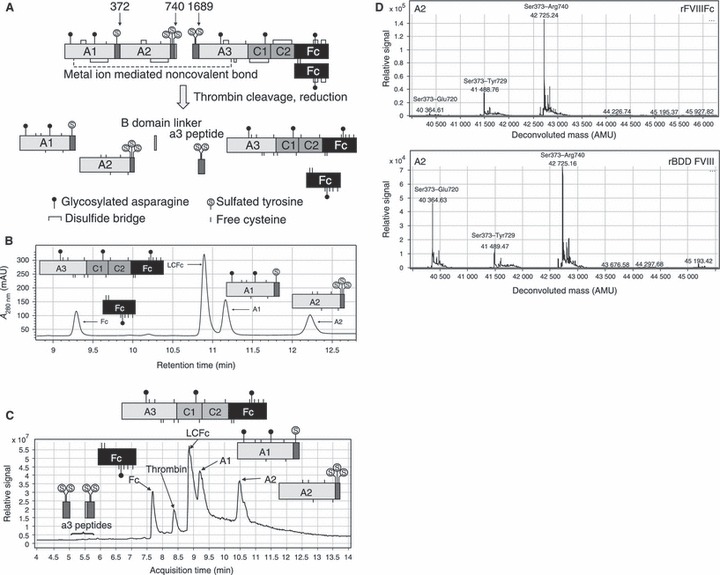
Thrombin mapping of recombinant factor VIII–Fc fusion protein (rFVIIIFc) by liquid chromatography–UV and liquid chromatography–mass spectrometry (MS). (A) Schematic of rFVIIIFc and thrombin cleavage products. (B) Liquid chromatography–UV map of rFVIIIFc after thrombin cleavage; major digestion products are indicated. (C) Liquid chromatography–MS map (total ion chromatogram) of rFVIIIFc after thrombin cleavage; major digestion products are indicated. (D) Deconvoluted mass spectra of the A2 domain of rFVIIIFc and recombinant B-domain-deleted FVIII (rBDD FVIII). Major products and their cognate masses are indicated, and correspond to thrombin-cleaved A2 domain (Ser373–Arg740) and two truncated products, Ser373–Tyr729 and Ser373–Glu720. AMU, atomic mass units; LC, light chain; mAU, milli absorbance units.

The rFVIIIFc structure was further analyzed by thrombin cleavage, reduction, and analysis by liquid chromatography–UV and liquid chromatography–MS. rFVIIIFc is cleaved by thrombin after three arginines, at positions 372, 740, and 1689, and this, under reducing conditions, generates four major peptides (Fig. 2A) that can be detected by UV absorbance (Fig. 2B), corresponding to the following segments of the protein: Fc (peak 1), LCFc (peak 2), and the A1 and A2 domains from the HC (peaks 3 and 4, respectively).

Analysis of the thrombin digestion of rFVIIIFc with HPLC–MS provided further detailed information on the four main domains, as well as the approximately 6-kDa a3-related peptides, and this was compared with the results of analysis of ReFacto, a CHO-derived rBDD FVIII protein, with the same methods. As expected, the total ion current chromatogram of rFVIIIFc (Fig. 2C) was similar to the UV chromatogram (Fig. 2B). Five of the expected six products (Fig. 2A) could be detected by liquid chromatography–MS, including two forms of the a3 acidic region generated from the processed and SC isoforms, as well as the thrombin used for the digestion. An additional truncated form of the a3 acidic region was also observed, and is described more fully below. rBDD FVIII yielded a similar total ion current chromatogram, but without the free Fc chain and with a different mass for the LC than that for the rFVIIIFc LCFc, which was consistent with the lack of an Fc region (data not shown).

Owing to the heterogeneity of glycosylation over much of the protein, the deconvoluted mass spectra for the A1, LCFc and Fc regions are complex; therefore, the identities of all of the molecular ions have not been established. However, the observed masses for the three major peaks from the Fc region were found to match the G0, G1 and G2 isoforms found on IgG molecules, which correspond to biantennary oligosaccharides terminating in no, one or two galactose residues (Fig. S2A; Table S1). The deconvoluted mass spectra of the a3-related peptides and the A2 domain provide the most definitive data, as there was no heterogeneity in the post-translational modifications in these regions, allowing the expected masses to be identified unambiguously.

The 6-kDa N-terminal peptide released from the LC after cleavage at Arg1689 is predicted to comprise the a3 acidic region (Glu1649–Arg1689) if derived from the processed isoform, and the 14 amino acid truncated B domain fused to the a3 acidic region if derived from the SC isoform. Both rFVIIIFc and rBDD FVIII were found to contain both forms of the a3 region, proportional to the expected levels based on SDS-PAGE analysis (Fig. S2B–E; Table S2). In addition, both proteins contained a truncated form of the a3 region, which corresponded to Asp1658–Arg1689, as has been reported for other FVIII products [3,10], although this was more abundant in rBDD FVIII than in rFVIIIFc.

The A2 domain contains three potential tyrosine sulfation sites, but no glycosylation sites that could result in complex heterogeneity, and therefore the exact masses of this region can be calculated (Table S3). In addition to the primary expected peak in the deconvoluted mass spectrum of rFVIIIFc correlating with the mass of the Ser373–Arg740 sequence (Fig. 2D), two additional forms were identified corresponding to known truncations of the FVIII HC [4], correlating with an A2 domain truncated at Glu720 and Tyr729. These reported truncated forms also were observed directly in the deconvoluted spectrum of the rBDD FVIII A2 domain (Fig. 2D). Both the rFVIIIFc and the rBDD FVIII A2 domains contained similar relative amounts of the form truncated at Tyr729, whereas the rBDD FVIII A2 domain contained a notably greater level of the form truncated at Glu720 than the rFVIIIFc A2 domain (Fig. 2D).

The primary sequence of rFVIIIFc was confirmed by peptide mapping with LysC digestion followed by UV and MS detection. Of the 99 theoretical peptides produced from rFVIIIFc, 81 were detected, corresponding to 98% of the total sequence (Table S4). The post-translational modifications of rFVIIIFc were also characterized with this method. FVIII contains six potential tyrosine sulfation sites, which corresponded to positions 346, 718, 719, 723, 1664, and 1680. Fully sulfated peptides that corresponded to these six sites were found with trace amounts of non-sulfated peptide corresponding to position 1680, as assessed by integration of the total ion chromatogram in the mass spectra, and there were no detectable non-sulfated peptides corresponding to the other positions. BDD FVIII also contains six potential N-glycosylation sites, four of which have been reported to be glycosylated in rFVIII products [4]. Consistent with this, rFVIIIFc was found to have the same four sites glycosylated; Asn239 and Asn2118 were found to contain high-mannose structures, whereas Asn41 and Asn1810 were found to contain more complex carbohydrates, similar to those found on rBDD FVIII. The Fc region N-linked glycosylation was found to match the G0, G1 and G2 isoforms found with the thrombin map by lquid chromatography–MS. FVIII has been reported to have O-glycosylation sites at Ser741 and Ser743 that are partially occupied [4], and this was found to be the case with rFVIIIFc as well.

### Activity by one-stage clotting (activated partial thromboplastin time [APTT]) and chromogenic assays

FVIII activity was measured with two different assays: the one-stage clotting assay (FVIII-specific APTT assay) and an FVIII chromogenic assay. The average specific activity from 14 separate batches of rFVIIIFc was found to be 8460 ± 699 IU mg^−1^ by the one-stage clotting assay and 9348 ± 1353 IU mg^−1^ by the chromogenic assay, which corresponded to 1861 ± 154 and 2057 ± 298 IU nmol^−1^, respectively. For comparison, the lot of ReFacto used in these studies was measured in the same chromogenic assay, and found to have a specific activity of 10950 IU mg^−1^, corresponding to 1862 IU nmol^−1^.

### Activity in FXase complex

**Figure 3:**
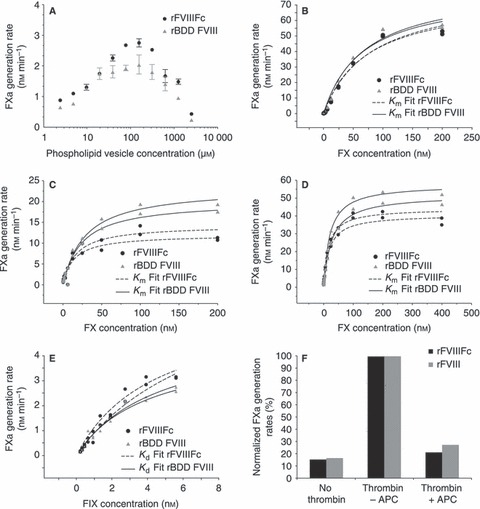
Characterization of recombinant factor VIII–Fc fusion protein (rFVIIIFc) and rFVIII activity in the FXase complex. The FXase complex was assembled with either rFVIIIFc or recombinant B-domain-deleted FVIII (rBDD FVIII) as indicated in Materials and methods, and FXa generation rates were determined. Data were generated by varying either phospholipid concentration (A) or FX concentration on phospholipid vesicles (B), resting platelets (C), or activated platelets (D), or FIXa concentration (E). Corresponding kinetic parameters are shown in Table 1. FXa generation rates were determined for FXase complexes formed with rFVIIIFc or rFVIII without thrombin activation, with thrombin activation, or with thrombin activation and activated protein C (APC) treatment, and normalized to rates with thrombin activation (F).

FVIII activity was assessed in the context of the FXase complex, after activation with thrombin followed by incubation with FIXa, phospholipids and FX in the presence of calcium, and measured by cleavage of an FXa substrate. The FXa generation rates were determined as a function of varying phospholipid concentrations for rFVIIIFc and rBDD FVIII (Fig. 3A), with phospholipid vesicles (25% PS/75% PC). The two proteins were found to have similar activity profiles, with peak activity at approximately 156 μm phospholipids.

The FXa generation rates were then determined as a function of varying FX concentrations on PS/PC, resting platelets, or activated platelets, and affinity (*K*_m_) and maximum reaction rate (*V*_max_) values were calculated (Fig. 3B–D). The activity profiles for rFVIIIFc and rBDD FVIII were found to be similar, with similar *K*_m_ and *V*_max_ (Table 1A), with either synthetic phospholipid vesicles or platelets as a phospholipid source. Finally, the FXa generation rate was determined as a function of varying FIXa concentrations on phospholipid vesicles (Fig. 3E). The activity profiles appeared similar, with similar *K*_d_ and *V*_max_ (Table 1B).

**Table 1:** (A) Factor X interactions with FXase complexes assembled with either recombinant FVIII–Fc fusion protein (rFVIIIFc) or recombinant B-domain-deleted FVIII (rBDD FVIII) on various phospholipid sources as determined by FXa generation assays. (B) FIXa interactions with FXase complexes assembled with either rFVIIIFc or rBDD FVIII as determined by FXa generation assays

Phospholipid source	Protein	*K*_m_ (nm)	*V*_max_ (nm min^−1^)
(A)			
25% PS/75% PC vesicles	rFVIIIFc	90.9 ± 11.9	86.4 ± 10.7
rBDD FVIII	69.2 ± 11.8	86.5 ± 12.2
Resting platelets	rFVIIIFc	11.0 ± 2.75	0.39 ± 0.04
rBDD FVIII	13.6 ± 3.9	0.53 ± 0.04
Activated platelets	rFVIIIFc	11.3 ± 1.8	5.00 ± 0.56
rBDD FVIII	14.0 ± 2.3	6.10 ± 0.60
Protein	*K*_d_ (nm)	*V*_max_ (nm min^−1^)
(B)		
rFVIIIFc	5.5 ± 1.1	6.9 ± 0.7
rBDD FVIII	4.5 ± 0.6	5.7 ± 1.3

PC, phosphatidylcholine; PS, phosphatidylserine.

### Inactivation by APC

Once active, FVIIIa is inactivated by cleavage by APC and by dissociation of the A2 domain. rFVIIIFc and rBDD FVIII were both activated by thrombin, and incubated with APC for 90 min; the activity was then determined in an FXa generation assay (Fig. 3F). In the absence of thrombin activation, little FXa generation was detected, and this was increased significantly with thrombin digestion. Treatment with APC for 90 min led to a significant decrease in FXa generation rates, similar to what was found for non-activated samples, and these results were similar for rFVIIIFc and rBDD FVIII.

### Affinity for VWF

The affinities of rFVIIIFc and rBDD FVIII for human VWF were determined by SPR. Both rFVIIIFc and rBDD FVIII showed high affinity for VWF, with *K*_D_ values of 0.34 and 0.26 nm, respectively (Table 2). This difference in observed *K*_D_ values is attributable to a difference in the association rate constants (30% greater for rBDD FVIII) rather than to a difference in dissociation rate constants. The data agreed well with a 1 : 1 association model (Fig. S3).

**Table 2:** Affinity of recombinant factor VIII–Fc fusion protein (rFVIIIFc) and recombinant B-domain-deleted FVIII (rBDD FVIII) for von Willebrand factor (VWF) as determined by surface plasmon resonance

Analyte	*n*	*k*_a_ (m^−1^ s^−1^)	*k*_d_ (s^−1^)	*K*_D_ (m)
rFVIIIFc	6	(2.6 ± 0.4) × 10^6^	(8.9 ± 1.3) × 10^−4^	(3.4 ± 0.1) × 10^−10^
rBDD FVIII	6	(3.4 ± 0.1) × 10^6^	(8.7 ± 0.4) × 10^−4^	(2.6 ± 0.1) × 10^−10^

### Clotting activity by in vitro ROTEM

**Figure 4:**
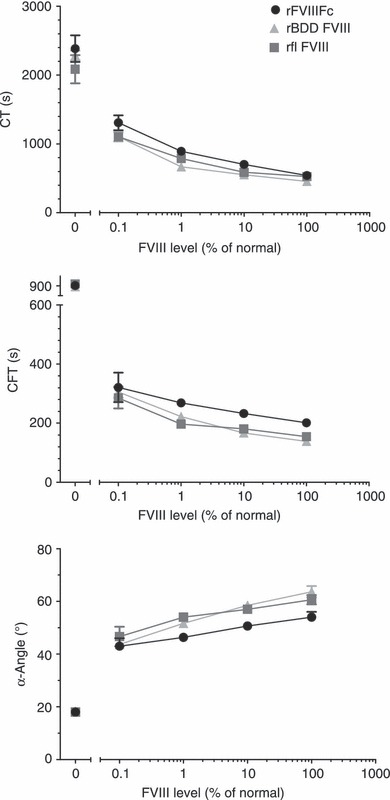
In vitro rotational thromboelastometry (ROTEM) data. ROTEM (non-activated thromboelastometry) results (mean ± standard deviation) for varying concentrations of Xyntha, Advate and recombinant factor VIII–Fc fusion protein (rFVIIIFc) spiked in pooled whole blood obtained from naïve mice with hemophilia A. Average clotting time (CT), clot formation time (CFT) and α-angle were determined. rBDD FVIII, recombinant B-domain-deleted FVIII; rflFVIII, recombinant full-length FVIII.

The clotting potency of rFVIIIFc was further explored with whole blood ROTEM over a range of concentrations, and compared with those of both rBDD FVIII (Xyntha) and recombinant full-length FVIII (rflFVIII; Advate). CT, CFT and the α-angle for the three proteins spiked in blood from mice with hemophilia A at escalating doses from 0.1% to 100% of normal FVIII levels (Fig. 4). In the wide range of 0.1–100% of normal, CT and CFT were comparable among rFVIIIFc, rBDD FVIII, and rflFVIII. The α-angle was only significantly different (*P* < 0.05) between rFVIIIFc and rBDD FVIII at 10%.

### Clotting activity by ex vivo ROTEM

**Figure 5:**
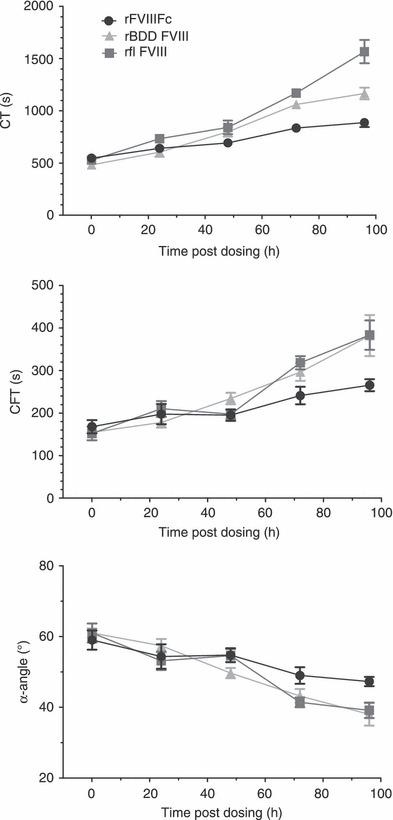
Ex vivo rotational thromboelastometry (ROTEM) data. ROTEM (non-activated thromboelastometry) results (mean ± standard deviation) from mice with hemophilia A following a single intravenous administration of 50 IU kg^−1^ recombinant B-domain-deleted factor FVIII (rBDD FVIII), recombinant full-length FVIII (rflFVIII) or recombinant FVIII–Fc fusion protein (rFVIIIFc) at 5 min, 24 h, 48 h, 72 h and 96 h after dosing. Clotting time (CT), clot formation time (CFT) and α-angle were determined.

The pharmacodynamics of rFVIIIFc, as measured with ROTEM, were compared with those of rBDD FVIII and rflFVIII after a single intravenous injection into mice with hemophilia A. CT, CFT and the α-angle were determined for samples taken from 5 min to 96 h after dosing (Fig. 5). At 5 min, all were similarly effective, resulting in similar CT, CFT, and α-angle (Fig. 5). However, rFVIIIFc showed a significantly improved (*P* < 0.05) CT at 72 and 96 h, and significantly improved CFT and α-angle at 96 h (Fig. 5), relative to rBDD FVIII and rflFVIII.

## Discussion

rFVIIIFc utilizes the naturally occurring FcRn recycling pathway to extend the half-life of FVIII and provide prolonged time in the circulation. rFVIIIFc is expressed as a monomeric Fc fusion protein, a configuration that has been shown to provide enhanced pharmacokinetic and pharmacodynamic properties in vivo with this molecule [6], erythropoietin [11], FIX [12], and interferon-β [13,14].

Several methods were used to confirm the primary sequence and post-translational modifications of FVIII, including peptide mapping after LysC digestion and thrombin map analysis by liquid chromatography–UV and liquid chromatography–MS. These analyses confirmed that rFVIIIFc contains the expected sequence based on the DNA coding sequence and post-translational modifications, such as N-linked glycosylation and tyrosine sulfation.

In addition, several functional assays were performed that demonstrated that rFVIIIFc has similar in vitro activity to rBDD FVIII. The specific activity of rFVIIIFc was found to be similar to that of rBDD FVIII on a molar basis by chromogenic activity (2057 IU nmol^−1^ for rFVIIIFc as compared with the 1549–2329 IU nmol^−1^ range listed for ReFacto, as specified in the product insert, and determined to be 1862 IU nmol^−1^ for ReFacto in this same assay). Unlike previous reports on discrepancies between the specific activity for one rBDD FVIII determined with chromogenic and one-stage clotting assays [15], the specific activity for rFVIIIFc appeared similar with these two assays, which is similar to more recent findings with another rBDD FVIII as well as full-length FVIII molecules [16]. In addition to the standard activity assays, several in vitro assays related to FVIII coagulation activity were performed within the context of the FXase complex to compare the interactions of rFVIIIFc and rBDD FVIII with other components of the coagulation cascade. In all analyses, the two proteins showed similar properties, which demonstrated that the activity of the FVIII moiety of rFVIIIFc is not compromised as a result of the Fc fusion. Importantly, this was found to be the case both when activated human platelets were used as a phospholipid source and when synthetic vesicles were used.

Human VWF is a large plasma glycoprotein that circulates in a high-affinity non-covalent complex with 95–98% of endogenous FVIII, and is important for the maintenance of appropriate plasma levels of FVIII in vivo [17]. The interaction of rFVIIIFc with VWF was examined with SPR, and the affinity was found to be in the sub-nanomolar range. In normal human plasma, the VWF concentration is in 50-fold excess of FVIII. The physiologic and clinical significance of the modestly reduced affinity for VWF of rFVIIIFc (∼ 30% weaker than that of rBDD FVIII) is probably not relevant. In fact, in vivo studies have indicated that the half-life of rFVIIIFc is significantly prolonged as compared with either recombinant full-length or BDD FVIII [6,7], despite this slightly weaker affinity for VWF in vitro, and therefore the FVIII–VWF interaction appears to be functional in vivo.

rFVIIIFc was found to have a higher level (15–25%) of SC rFVIIIFc than other rBDD FVIIIs [4,10], which contain 3–5% SC FVIII. The SC FVIIIFc isoform could be converted to fully processed rFVIIIFc by cotransfection with different processing enzymes, although some appeared to degrade the HC as well. This SC FVIIIFc, which differs from processed rFVIIIFc by a single peptide bond between Arg1648 and Glu1649, was purified and characterized in all of the biochemical assays described above, and found to be similar to rFVIIIFc (manuscript in preparation). Note that thrombin digestion removes the remaining B-domain region during FVIII activation, and thus converts SC FVIII to the same final products as rBDD FVIII and rflFVIII; therefore, the activated versions of all of these forms are indistinguishable. Further study will be required to determine whether the SC FVIII molecule shows greater stability because of the covalent linkage of the HC and LC, potentially reducing chain dissociation in the circulation.

In addition to these in vitro studies, the functional activity of rFVIIIFc was assessed with ROTEM, and rFVIIIFc showed similar clotting potency as both rBDD FVIII and rflFVIII in vitro. Importantly, rFVIIIFc showed equivalent effectiveness to rBDD FVIII and rflFVIII in acute clot formation ex vivo immediately following treatment of mice with hemophilia A. Furthermore, rFVIIIFc showed prolonged improvement in clot formation relative to rBDD FVIII and rflFVIII following a single intravenous injection in mice with hemophilia A, which is consistent with the extended pharmacokinetics of rFVIIIFc in hemophilic mice and dogs [6].

In conclusion, these data demonstrate that rFVIIIFc has full activity as compared with other FVIII products in vitro and ex vivo, and has prolonged activity in the circulation. Prolonged activity as compared with rflFVIII was shown recently in a phase 1/2 human clinical trial [7]. Repeat-dose clinical studies with rFVIIIFc are in progress to evaluate the acute efficacy and prolonged protection from bleeding observed in these and other preclinical studies.

## Supporting Information

**Figure S1**Amino acid sequence of rFVIIIFc. **Figure S2**. Thrombin mapping of rFVIIIFc andrBDD FVIII by liquid chromatography/mass spectrometry: Deconvolutedmass spectra of the Fc fragment of rFVIIIFc (A), and the spectrafor the 6 kDa light-chain N-terminus peptide fragment of rFVIIIFc(B and C) and rBDD FVIII (D and E). **Figure S3** Surface plasmon resonance sensorgrams that depict the interactions of (A) rFVIIIFc and (B) rBDD FVIII with immobilized hVWF. **Table S1**. Identification of Fc containingdifferent *N-*linked glycans of rFVIIIFc. **Table S2**. Identification of 6 kDa LCN*-*terminus peptide of rFVIIIFc. **Table S3**. Identification of heterogenities ofA2 domain of rFVIIIFc. **Table S4**. List of the major peptides ofrFVIIIFc after LysC digest and MS analysis.
