# Supplementary material for: Biochemical and functional characterization of a recombinant monomeric factor VIII–Fc fusion protein
Source: J Thromb Haemost. 2013 Jan;11(1):132–41. doi: 10.1111/jth.12076 (PMC3588154; doi:10.1111/jth.12076)
Supplement: Supplementary file 1 — Figure S1Amino acid sequence of rFVIIIFc. Figure S2. Thrombin mapping of rFVIIIFc andrBDD FVIII by liquid chromatography/mass spectrometry: Deconvolutedmass spectra of the Fc fragment of rFVIIIFc (A), and the spectrafor the 6 kDa light-chain N-terminus peptide fragment of rFVIIIFc(B and C) and rBDD FVIII (D and E). Figure S3 Surface plasmon resonance sensorgrams that depict the interactions of (A) rFVIIIFc and (B) rBDD FVIII with immobilized hVWF. Table S1. Identification of Fc containingdifferent N-linked glycans of rFVIIIFc. Table S2. Identification of 6 kDa LCN-terminus peptide of rFVIIIFc. Table S3. Identification of heterogenities ofA2 domain of rFVIIIFc. Table S4. List of the major peptides ofrFVIIIFc after LysC digest and MS analysis. [file jth0011-0132-sd1.pdf]

Supporting Information: Biochemical and functional characterization of rFVIIIFc (Peters RT et al)

Materials and Methods

Cloning, expression, and purification of rFVIIIFc

The coding sequence of human B-domain deleted (BDD) clotting factor VIII (FVIII) (S743 to Q1638 fusion) and human immunoglobulin G1 (IgG1) Fc (hinge, CH2, and CH3 domains, D221 to G456, Kabat numbering) were obtained by a combination of reverse transcription–polymerase chain reactions (RT-PCR) and PCR and fused with no intervening linker. For expression of the Fc chain alone, the mouse Igκ light chain signal sequence was created with synthetic oligonucleotides and added to the Fc coding sequence using PCR, and the two coding sequences were expressed from the same plasmid. HEK293H cells (Invitrogen, Life Technologies Corporation, Grand Island, NY, USA) were transfected using Lipofectamine™ transfection reagent (Invitrogen), and a stable cell line was selected. Cells were grown in serum-free suspension culture, and rFVIIIFc protein was purified from clarified harvest media using a three-column purification process, including a FVIII-specific affinity purification step [1], followed by a combination of anion exchange columns and a hydrophobic interaction column. Nanofiltration and a detergent hold (virus inactivation) step also were employed as part of the purification process.

For transient transfection experiments (data in Figures 1C, 1D, and 1E), a second plasmid was constructed expressing rFVIIIFc as a single polypeptide chain in which the C-terminus of the first Fc region is connected to the N-terminus of the second Fc region with a 20 amino acid Gly/Ser flexible linker (corresponding to 4 repeats of GGGGS). This plasmid produces only the

rFVIIIFc monomer without free Fc. Coding sequences for processing enzymes were obtained by similar techniques and cotransfected with either Lipofectamine or polyethyleneimine (PEI) using standard protocols. Sodium dodecyl sulfate polyacrylamide gel electrophoresis (SDS-PAGE) and Western analysis were performed using standard techniques.

Thrombin and lysyl endopeptidase peptide mapping

FVIII samples were passed through Detergent-OUTDTG-100X columns (GBioscience, Maryland Heights, MO, USA) for the removal of Tween according to manufacturer's directions. 30 to 60 µg of FVIII sample was incubated for 30 min at 37ºC with 0.033 U thrombin (Hematologic Technologies, Cat. No. HCT-0020)/µg FVIII sample in final buffer of 50 mM Tris-HCl, 150 mM NaCl, 2 mM $CaCl_2$, 5% glycerol, pH 7.4 in a final volume of 200 µL. Samples were reduced by adding 60 µL 2-mercaptoethanol (J.T. Baker, Cat. No. 4049-00) and incubated at 95ºC for 5 min. Samples were then analyzed by either reversed phase high-performance liquid chromatography (RP-HPLC) on a POROS R1/10 column (Applied Biosystems, Life Technologies Corporation, Carlsbad, CA, USA) with ultraviolet detection (RP-HPLC/UV, detection wavelength 214 nm) or mass spectrometry detection (RP-HPLC/MS; Agilent 1200 coupled to an Agilent 6210TOF mass spectrometer) using mobile phase A (0.1% formic acid in water) and mobile phase B (0.1% formic acid in acetonitrile) as follows: 10%B from 0 to 3 min, gradients to 80% B at 18.0 min, held at 80% B to 21.0 min, then a gradient to 100% B 21.5 min, with a flow rate of 1.0 mlL/min, column temperature of 60ºC. For MS detection, the following parameters were used: Electrospray voltage, 3kV; Gas temperature, 250°C; Fragmentor voltage, 180V; Drying gas, 11 L/min; Nebulizer, 55 Psi; Skimmer, 65V; Oct 1 RF Vapp, 250V.

Peptide sequence was also confirmed with lysyl endopeptidase peptide mapping as follows. FVIII samples (50-70 µg) was dried down in a speed vac. Samples were then reduced by reconstituting with 99 µL of denaturing buffer (6M guanidine, 2.5 mM EDTA, 50 mM Tris-HCl, pH 7.5) and 1 µL of 1.0 M DTT solution, and incubating at 25ºC for 1h. Samples were digested with LysC by adding 200 µL of 50 mM Tris-HCl/1 mM EDTA followed by 10 µL of 0.5 µg/µL LysC and incubated at 25ºC for 16-20 hrs. 2 µL of 10% TFA was added into 100 µL digest to terminate the digestion. The samples were frozen in liquid nitrogen and stored at -20ºC until analysis by RP-HPLC/MS (Thermo Finnigan LTQ-XL-ETD, Thermo Fisher Scientific Inc., Waltham, MA, USA). 100 µL of each sample was injected on to a YMC™ ODS-A, 5µm, 120 Å, 2.0x250 mm column (Waters, Cat. No. AA12S052502WT) maintained at 55 ºC with a flow rate of 0.25 mL/min, with UV detection at 214 nm, and run on a gradient with mobile phase A (0.1% TFA in water) and mobile phase B (90% acetonitrile, 0.1% TFA in water), as follows: 0%B from 0 to 8 min, gradients to 15% B at 38.0 min, 24% B at 88.0 min, 26.5% B at 108.0 min, 30%B at 128.0 min, 45% B at 198 min, and 100% B at 216.0 min. The LTQ-XL-ETD ion trap mass spectrometer was connected in-line after the photodiode array detector, and operated in the electrospray ionization (ESI) mode. "Triple Play" (sequential full scan, zoom scan, and MS/MS scan) and data dependent MS/MS scan utilizing dynamic exclusion were employed. The MS/MS data was searched against Sequest database in BioworksBrowser 3.3.1 SP1 for peptide sequencing. The following parameters were used: Sheath gas flow rate (arbitrary), 50; Electrospray voltage, 4.0 kV; Capillary temperature, 200ºC; Capillary voltage, 49 V; Tube lens, 95 V. Peaks were identified by molecular ion masses, which were in good agreement with the corresponding theoretical molecular ion mass. The FVIIIFc sequence coverage is calculated to

be 98% based on identified peptides over the total sequence, and only seventeen very hydrophilic small peptides were not detected.

Preparation of phospholipid vesicles and platelets

Phospholipids were purchased from Avanti Polar Lipids (Cat# 840032C for phosphatidylserine (PS) and 840051C for phosphatidylcholine (PC), in chloroform), mixed to 25% PS/75% PC, lyophilized, resuspended to 20 mM total phospholipids in 1 ml of 50 mM Tris pH 7.4, 150 mM NaCl, incubated with shaking at 68ºC for 30 min, and phospholipid vesicles prepared by extrusion using the Avanti Polar Lipids Mini-Extruder on a 68ºC pre-heated block according to the manufacturer's directions, extruded 12 times through a 100 nm pore size filter. Platelets were purified by gel filtration (Sepharose CL-2B column) from platelet rich plasma obtained from normal human donors, eluted in a final buffer of 15 mM HEPES, 138 mM NaCl, 2.7 mM KCl, 1 mM $MgCl_2$, 5.5 mM dextrose, pH 7.4.

Activity in Xase complex

Methods for determining activity in Xase complex were performed as described [2]. , In general FVIII was first activated with α-thrombin for 5 minutes, then stopped with hirudin and mixed with FIXa in the presence of Ca^{2+} and synthetic phospholipid vesicles (25% phosphatidylserine/75% phosphatidylcholine) or platelets. FVIIIa and FIXa interact in the presence of a phospholipid surface and calcium ions to form an active Xase complex that mediates the conversion of FX into FXa through proteolytic processing. In turn, FXa cleaves an FXa-specific chromogenic or fluorogenic substrate. The cleaved substrate is chromogenic (or fluorogenic where platelets are used) and therefore the amount of cleaved substrate in a solution

is indicative of the amount of FXa generated. This is quantified by measuring the absorbance of the solution at 405 nm (or fluorescence with excitation at 360 nm and emission at 460 nm) and the kinetic parameters determined. Parameters for all assays are expressed as mean ± standard deviation, from three replicate runs, each in duplicate, for all assays with phospholipids, and with two replicate runs, each in duplicate, for platelet assays.

All reactions were carried out at room temperature. For Fig 3A, final Xase reaction contained 0.5 nM activated FVIII sample with 0.5 nM FIXa and 100 nM FX, in 50 mM Tris pH 7.4, 100 mM NaCl, 5 mM $CaCl_2$, 0.2% BSA, with 0.5 mM Perfachrome FXa 6034 substrate, and 11 concentrations of 25% PS/75% PC (2-fold dilutions, 2500 to 2.4 µM final concentration). For Fig 3B, the final Xase reaction contained 2 nM FVIIIa, 1 nM FIXa, 100 µM phospholipids (25% PS/75% PC), in 50 mM Tris pH 7.4, 100 mM NaCl, 5 mM $CaCl_2$, 0.2% BSA, and 0.5 mM Pefachrome FXa 6034, and various FX concentrations ranging between 0.39 nM and 400 nM, in 2-fold increments. For Fig 3C, the final Xase reaction contained 2 nM FVIIIa, 1 nM FIXa, final concentration of 3 x 10^7 platelets/ml in 50 mM Tris pH 7.4, 100 mM NaCl, 5 mM $CaCl_2$, 0.2% BSA, and 0.15 mM PefaFluor FXa, and various FX concentrations ranging between 0.4 nM and 400 nM. For Fig 3D, the reaction was carried out under the same conditions, but the platelets were first activated by incubating with 50 µg/mL SFLLRN peptide 10 µM ADP for 15 min at 37°, and then added to a final concentration of 3 x 10^7 platelets/ml. For Fig 3E, the final Xase reaction contained 0.2 nM FVIIIa, 100 nM FX, 100 µM phospholipids (25% PS/75% PC), in 50 mM Tris pH 7.4, 100 mM NaCl, 5 mM $CaCl_2$, 0.2% BSA, and 0.5 mM Pefachrome FXa 6034, and 11 different FIXa concentrations ranging between 0.22 nM and 8 nM.

For activated protein C inactivation in Figure 3F, thrombin-activated FVIII samples (at 250 nM) were treated with hirudin and then activated protein C (at 25 μM) for 90 minutes before assaying Xase activity under the same conditions as for Fig 3B, above, utilizing 100 nM FX. FXa generation rates were obtained from the mean of duplicate runs and normalized to activity levels with the addition of thrombin.

Surface plasmon resonance analysis of von Willebrand Factor binding

All analyses were performed on a Biacore T100 instrument (GE Healthcare, Piscataway, NJ, USA) at 25°C. Approximately 500 resonance units of purified FVIII-free plasma-derived human von Willebrand Factor (hVWF; Haematologic Technologies, Inc, Essex Junction, VT, USA) were immobilized at each of three flow cells of a CM5 biosensor chip (GE Healthcare, Piscataway, NJ, USA) by standard amine coupling at pH 5, and residual-activated sites were subsequently blocked with ethanolamine. rFVIIIFc and rBDD FVIII analyte samples were applied sequentially at 25 μL/min in single-cycle kinetics mode [3] at concentrations of 0.13, 0.32, 0.8, 2.0, and 5.0 nM, each for 240 seconds followed by a terminal dissociation phase of 1200 seconds. Binding and dissociation steps were performed in a buffer that consisted of 6.7 mM L-histidine, 5.3 mM $CaCl_2$, 205 mM NaCl, 1.3% sucrose, and 0.013% polysorbate 20, pH 7.0. The hVWF coupled chip surface was regenerated between cycles with a solution of 0.6 M NaCl and 0.35 M $CaCl_2$. Raw data were processed by double reference subtraction, and kinetic parameters were derived from a 1:1 binding model that yielded values for χ^2/R_{max} of 0.02 or less.

Tables:

Table S1. Identification of Fc containing different *N*-linked glycans of rFVIIIFc

Fc glycoforms	Theoretical mass* (Da)	Observed mass (Da)
Fc (G0)	26865.74	26866.05
Fc (G1)	27027.79	27028.10
Fc (G2)	27189.84	27190.60

* The mass is calculated based on the Fc primary sequence and known individual glycan composition

Table S2. Identification of 6 kDa LC N-terminus peptide of rFVIIIFc .

Sequence	Number of sulfates	Theoretical mass (Da)	Ions observed
^{741}S-R$^{795(1689)}$	2	6779.09	1695.5 (4+), 1356.6 (5+), 1130.7 (6+)
$^{755(1649)}$E-R$^{795(1689)}$	2	5102.18	1701.4 (3+), 1276.3 (4+)
$^{764(1658)}$D-R$^{795(1689)}$	2	4073.02	1358.3 (3+), 1019.0 (4+)

Table S3. Identification of heterogenities of A2 domain of rFVIIIFc

A2 primary sequence	Number of sulfates	Theoretical mass (Da)	Observed mass (Da)
^{373}S-E^{720}	2	40364.86	40364.61
^{373}S-Y^{729}	3	41488.90	41488.76
^{373}S-R^{740}	3	42725.34	42725.24

Table S4. List of the major peptides of rFVIIIFc after LysC digest and MS analysis. Amino acid numbering is based on the contiguous sequence listed in Fig S1, below. FVIII peptides: in Bold; Fc peptides: in *Italic*. For glycopeptides, the observed mass was listed as "PTM" since no single mass was detected because of the heterogeneity; the listed theoretical molecular ion masses for these glycopeptides are only based on amino acid sequences. Y(s) indicates a sulfated tyrosine residue.

**Peptide number**	**From-To**	**$[M+H]^+$ (theoretical)**	**$[M+H]^+$ (observed)**	**Sequence**
**L1**	1-36	4198.8	4198.9	ATRRYYLGAVELSWDYMQSDLGELPVDARFPPRVPK
**L2 (N-Glyco)**	37 - 47	1289.5	PTM	SFPF**NTS**VVYK
**L3**	48 - 48	147.2	**not detected**	K
**L4**	49 - 63	1796.1	1795.8	TLFVEFTDHLFNIAK
**L5**	64 - 89	2896.5	2896.0	PRPPWMGLLGPTIQAEVYDTVVITLK
**L6**	90 - 107	1984.3	1983.8	NMASHPVSLHAVGVSYWK
**L7**	108 - 123	1814.8	1814.6	ASEGAEYDDQTSQREK
**L8**	124 - 127	506.5	**not detected**	EDDK
**L9**	128 - 142	1719.0	1718.6	VFPGGSHTYVWQVLK
**L10**	143 - 166	2654.0	2654.2	ENGPMASDPLCLTYSYLSHVDLVK
**L11**	167 - 186	2030.4	2030.2	DLNSGLIGALLVCREGSLAK
**L12**	187 - 188	276.3	**not detected**	EK
**L13**	189 - 194	727.8	727.5	TQTLHK
**L14**	195 - 206	1399.7	1399.6	FILLFAVFDEGK
**L15**	207 - 213	874.9	875.0	SWHSETK
**L16**	214 - 230	1918.1	1917.8	NSLMQDRDAASARAWPK
**L17 (N-Glyco)**	231 - 251	2353.8	PTM	MHTVNGYV**NRS**LPGLIGCHRK
**L18**	252 - 325	8439.8	8440.6	SVYWHVIGMGTTPEVHSIFLEGHTFLVRNHRQASLEISPITFLT AQTLLMDLGQFLLFCHISSHQHDGMEAYVK
**L19**	326 - 338	1532.8	1533.1	VDSCPEEPQLRMK
**L20**	339 - 376	4476.6	4477.3	NNEEAED**Y(s)**DDDLTDSEMDVVRFDDDNSPSFIQIRSVAK
**L21**	377 - 377	147.2	**not detected**	K
**L22**	378 - 380	381.5	**not detected**	HPK

**L23**	381 - 408	3354.6	3355.0	TWVHYIAAEEEDWDYAPLVLAPDDRSYK
**L24**	409 - 422	1631.8	1631.6	SQYLNNGPQRIGRK
**L25**	423 - 424	310.4	**not detected**	YK
**L26**	425 - 425	147.2	**not detected**	K
**L27**	426 - 437	1508.7	1508.6	VRFMAYTDETFK
**L28**	438 - 466	3184.7	3185.5	TREAIQHESGILGPLLYGEVGDTLLIIFK
**L29**	467 - 493	3213.6	3214.0	NQASRPYNIYPHGITDVRPLYSRRLPK
**L30**	494 - 496	303.4	**not detected**	GVK
**L31**	497 - 499	397.5	397.3	HLK
**L32**	500 - 510	1276.5	1276.4	DFPILPGEIFK
**L33**	511 - 512	310.4	**not detected**	YK
**L33-L34**	511 - 523	1524.7	1524.6	YKWTVTVEDGPTK
**L34**	513 - 523	1233.4	1233.4	WTVTVEDGPTK
**L35**	524 - 556	3783.4	3784.2	SDPRCLTRYYSSFVNMERDLASGLIGPLLICYK
**L36**	557 - 570	1607.7	1607.4	ESVDQRGNQIMSDK
**L37**	571 - 659	10350.7	10351.2	RNVILFSVFDENRSWYLTENIQRFLPNPAGVQLEDPEFQASNIM HSINGYVFDSLQLSVCLHEVAYWYILSIGAQTDFLSVFFSGYTF K
**L38**	660 - 661	284.3	**not detected**	HK
**L37-L39**	571 - 707	15869.1	15869.6	RNVILFSVFDENRSWYLTENIQRFLPNPAGVQLEDPEFQASNIM HSINGYVFDSLQLSVCLHEVAYWYILSIGAQTDFLSVFFSGYTF KHKMVYEDTLTLFPFSGETVFMSMENPGLWILGCHNSDFRNR GMTALLK
**L39**	662 - 707	5272.1	5273.0	MVYEDTLTLFPFSGETVFMSMENPGLWILGCHNSDFRNRGMT ALLK
**L40**	708 - 713	638.7	638.4	VSSCDK
**L41**	714 - 733	2587.5	2587.0	NTGD**Y(s)Y(s)**EDS**Y(s)**EDISAYLLSK
**L42**	734 - 750	1912.1	1911.8	NNAIEPRSFSQNPPVLK
**L42 (O-glyco)**	734 - 750	1912.1	PTM	NNAIEPR**S**F**S**QNPPVLK
**L43 (single chain)**	751-779	3618.8	3619.3	RHQREITRTTLQSDQEEID**Y(s)**DDTISVEMK
**L43 (processed)**	755 - 779	3040.2	3040.6	EITRTTLQSDQEEID**Y(s)**DDTISVEMK
**L44-L45**	780 - 799	2570.6	2571.2	KEDFDI**Y(s)**DEDENQSPRSFQK
**L45**	781 - 799	2442.4	2441.4	EDFDI**Y(s)**DEDENQSPRSFQK
**L46**	800 - 800	147.2	**not detected**	K
**L47**	801 - 837	4293.8	4293.7	TRHYFIAAVERLWDYGMSSSPHVLRNRAQSGSVPQFK

**L48-L49**	838 - 910	8521.4	8521.0	KVVFQEFTDGSFTQPLYRGELNEHLGLLGPYIRAEVEDNIMVT FRNQASRPYSFYSSLISYEEDQRQGAEPRK
**L49**	839 - 910	8393.2	8393.8	VVFQEFTDGSFTQPLYRGELNEHLGLLGPYIRAEVEDNIMVTF RNQASRPYSFYSSLISYEEDQRQGAEPRK
**L50-51 (N-Glyco)**	911 - 919	1077.3	PTM	NFVKP**NET**K
**L52**	920 - 924	744.9	744.5	TYFWK
**L53**	925 - 933	1049.2	1048.6	VQHHMAPTK
**L54**	934 - 939	756.8	756.4	DEFDCK
**L55**	940 - 951	1444.6	1444.4	AWAYFSDVDLEK
**L56**	952 - 993	4668.3	4669.3	DVHSGLIGPLLVCHTNTLNPAHGRQVTVQEFALFFTIFDETK
**L57**	994 - 1019	3212.6	3213.4	SWYFTENMERNCRAPCNIQMEDPTFK
**L58**	1020 - 1073	6366.3	6367.0	ENYRFHAINGYIMDTLPGLVMAQDQRIRWYLLSMGSNENIHSI HFSGHVFTVRK
**L59**	1074 - 1074	147.2	**not detected**	K
**L60**	1075 - 1078	568.6	568.4	EEYK
**L61**	1079 - 1098	2303.7	2303.6	MALYNLYPGVFETVEMLPSK
**L62**	1099 - 1126	3146.7	3146.4	AGIWRVECLIGEHLHAGMSTLFLVYSNK
**L63**	1127 - 1155	3150.6	3151.3	CQTPLGMASGHIRDFQITASGQYGQWAPK
**L64**	1156 - 1171	1805.0	1804.8	LARLHYSGSINAWSTK
**L65**	1172 - 1178	907.0	906.6	EPFSWIK
**L66**	1179 - 1191	1420.8	1420.6	VDLLAPMIIHGIK
**L67**	1192 - 1198	788.9	**not detected**	TQGARQK
**L68**	1199 - 1216	2113.5	2113.2	FSSLYISQFIIMYSLDGK
**L69-70 (N-Glyco)**	1217 - 1242	2894.2	PTM	KWQTYRG**NST**GTLMVFFGNVDSSGIK
**L70 (N-Glyco)**	1218 - 1242	2767.0	PTM	WQTYRG**NST**GTLMVFFGNVDSSGIK
**L71**	1243 - 1289	5446.5	5446.9	HNIFNPPIIARYIRLHPTHYSIRSTLRMELMGCDLNSCSMPLGM ESK
**L72**	1290 - 1313	2625.9	2625.4	AISDAQITASSYFTNMFATWSPSK
**L73**	1314 - 1333	2343.7	2344.6	ARLHLQGRSNAWRPQVNNPK
**L74**	1334 - 1342	1193.3	1193.4	EWLQVDFQK
**L75**	1343 - 1345	379.5	379.3	TMK
**L76**	1346 - 1355	990.1	989.7	VTGVTTQGVK
**L77**	1356 - 1364	1042.3	1042.2	SLLTSMYVK
**L78**	1365 - 1385	2470.7	2470.4	EFLISSSQDGHQWTLFFQNGK

**L79**	1386 - 1387	245.2	**not detected**	VK
**L80**	1388 - 1440	6141.0	6141.4	VFQGNQDSFTPVVNSLDPPLLTRYLRIHPQSWVHQIALRMEVL GCEAQDLYDK
L81	1441 - 1464	2507.0	2507.5	THTCPPCPAPELLGGPSVFLFPPK
L81-L82)	1441 - 1466	2732.3	2731.8	THTCPPCPAPELLGGPSVFLFPPKPK
L83	1467 - 1492	2900.3	2899.6	DTLMISRTPEVTCVVVDVSHEDPEVK
L84	1493 - 1506	1678.8	1678.6	FNWYVDGVEVHNAK
L85-L86 (N-glyco)	1507 - 1535	3462.9	PTM	TKPREEQY**NST**YRVVSVLTVLHQDWLNGK
L87	1536 - 1538	439.5	439.3	EYK
L88	1539 - 1540	250.3	**not detected**	CK
L89	1541 - 1544	447.5	**not detected**	VSNK
L90	1545 - 1552	839.0	839.0	ALPAPIEK
L91	1553 - 1556	448.5	448.4	TISK
L92	1557 - 1558	218.3	**not detected**	AK
L93	1559 - 1578	2312.6	2312.2	GQPREPQVYTLPPSRDELTK
L94	1579 - 1588	1105.3	1105.2	NQVSLTCLVK
L95	1589 - 1610	2545.7	2545.4	GFYPSDIAVEWESNGQPENNYK
L96	1611 - 1627	1875.1	1874.6	TTPPVLDSDGSFFLYSK
L97	1628 - 1632	575.7	575.4	LTVDK
L98	1633 - 1657	2989.3	2989.9	SRWQQGNVFSCSVMHEALHNHYTQK
L99	1658 - 1664	660.7	660.4	SLSLSPG

Figures

Fig S1. Amino acid sequence of rFVIIIFc. rFVIIIFc is a recombinant fusion protein comprised of a single molecule of B domain deleted human Factor VIII (BDD FVIII) fused to the dimeric human Fc region from IgG1 with no intervening linker sequence, generated as two polypeptide chains.

a) The rFVIIIFc chain amino acid sequence. The 14 amino acid linker from the truncated B domain is double underlined, with the SQ fusion (amino acids 743-744, corresponding to S743 and Q1638 of the full length FVIII sequence) and the intracellular processing site (R754, corresponding to R1648) indicated in bold. The Fc portion (wt IgG1, EU numbering D 221 to G 456) is indicated in italics and underlined.

1 ATRRYYLGAV ELSWDYMQSD LGELPVDARF PPRVPKSFPF NTSVVYKKTL

51 FVEFTDHLFN IAKPRPPWMG LLGPTIQAEV YDTVVITLKN MASHPVSLHA

101 VGVSYWKASE GAEYDDQTSQ REKEDDKVFP GGSHTYVWQV LKENGPMASD

151 PLCLTYSYLS HVDLVKDLNS GLIGALLVCR EGSLAKEKTQ TLHKFILLFA

201 VFDEGKSWHS ETKNSLMQDR DAASARAWPK MHTVNGYVNR SLPGLIGCHR

251 KSVYWHVIGM GTTPEVHSIF LEGHTFLVRN HRQASLEISP ITFLTAQTLL

301 MDLGQFLLFC HISSHQHDGM EAYVKVDSCP EEPQLRMKNN EEAEDYDDDL

351 TDSEMDVVRF DDDNSPSFIQ IRSVAKKHPK TWVHYIAAEE EDWDYAPLVL

401 APDDRSYKSQ YLNNGPQRIG RKYKKVRFMA YTDETFKTRE AIQHESGILG

451 PLLYGEVGDT LLIIFKNQAS RPYNIYPHGI TDVRPLYSRR LPKGVKHLKD

501 FPILPGEIFK YKWTVTVEDG PTKSDPRCLT RYYSSFVNME RDLASGLIGP

551 LLICYKESVD QRGNQIMSDK RNVILFSVFD ENRSWYLTEN IQRFLPNPAG

601 VQLEDPEFQA SNIMHSINGY VFDSLQLSVC LHEVAYWYIL SIGAQTDFLS

651 VFFSGYTFKH KMVYEDTLTL FPFSGETVFM SMENPGLWIL GCHNSDFRNR

701 GMTALLKVSS CDKNTGDYYE DSYEDISAYL LSKNNAIEPR SF**SQ**NPPVLK

751 RHQ**R**EITRTT LQSDQEEIDY DDTISVEMKK EDFDIYDEDE NQSPRSFQKK

801 TRHYFIAAVE RLWDYGMSSS PHVLRNRAQS GSVPQFKKVV FQEFTDGSFT

851 QPLYRGELNE HLGLLGPYIR AEVEDNIMVT FRNQASRPYS FYSSLISYEE

901 DQRQGAEPRK NFVKPNETKT YFWKVQHHMA PTKDEFDCKA WAYFSDVDLE

951 KDVHSGLIGP LLVCHTNTLN PAHGRQVTVQ EFALFFTIFD ETKSWYFTEN

1001 MERNCRAPCN IQMEDPTFKE NYRFHAINGY IMDTLPGLVM AQDQRIRWYL

1051 LSMGSNENIH SIHFSGHVFT VRKKEEYKMA LYNLYPGVFE TVEMLPSKAG

1101 IWRVECLIGE HLHAGMSTLF LVYSNKCQTP LGMASGHIRD FQITASGQYG

1151 QWAPKLARLH YSGSINAWST KEPFSWIKVD LLAPMIIHGI KTQGARQKFS

1201 SLYISQFIIM YSLDGKKWQT YRGNSTGTLM VFFGNVDSSG IKHNIFNPPI

1251 IARYIRLHPT HYSIRSTLRM ELMGCDLNSC SMPLGMESKA ISDAQITASS

1301 YFTNMFATWS PSKARLHLQG RSNAWRPQVN NPKEWLQVDF QKTMKVTGVT

1351 TQGVKSLLTS MYVKEFLISS SQDGHQWTLF FQNGKVKVFQ GNQDSFTPVV

1401 NSLDPPLLTR YLRIHPQSWV HQIALRMEVL GCEAQDLY*DK THTCPPCPAP*

1451 ELLGGPSVFL FPPKPKDTLM ISRTPEVTCV VVDVSHEDPE VKFNWYVDGV

1501 EVHNAKTKPR EEQYNSTYRV VSVLTVLHQD WLNGKEYKCK VSNKALPAPI

1551 EKTISKAKGQ PREPQVYTLP PSRDELTKNQ VSLTCLVKGF YPSDIAVEWE

1601 SNGQPENNYK TTPPVLDSDG SFFLYSKLTV DKSRWQQGNV FSCSVMHEAL

1651 HNHYTQKSLS LSPG

b) The Fc chain amino acid sequence.

1 DKTHTCPPCP APELLGGPSV FLFPPKPKDT LMISRTPEVT CVVVDVSHED

51 PEVKFNWYVD GVEVHNAKTK PREEQYNSTY RVVSVLTVLH QDWLNGKEYK

101 CKVSNKALPA PIEKTISKAK GQPREPQVYT LPPSRDELTK NQVSLTCLVK

151 GFYPSDIAVE WESNGQPENN YKTTPPVLDS DGSFFLYSKL TVDKSRWQQG

201 NVFSCSVMHE ALHNHYTQKS LSLSPG

Fig. S2. Thrombin mapping of rFVIIIFc and rBDD FVIII by liquid chromatography/mass spectrometry: Deconvoluted mass spectra of the Fc fragment of rFVIIIFc (A), and the spectra for the 6 kDa light-chain N-terminus peptide fragment of rFVIIIFc (B and C) and rBDD FVIII (D and E).

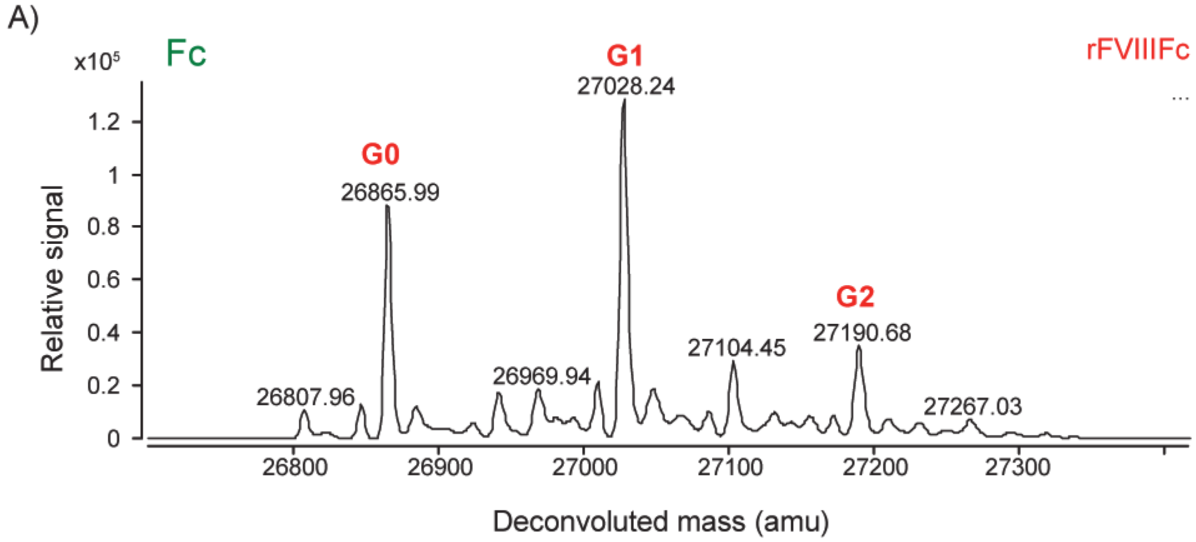

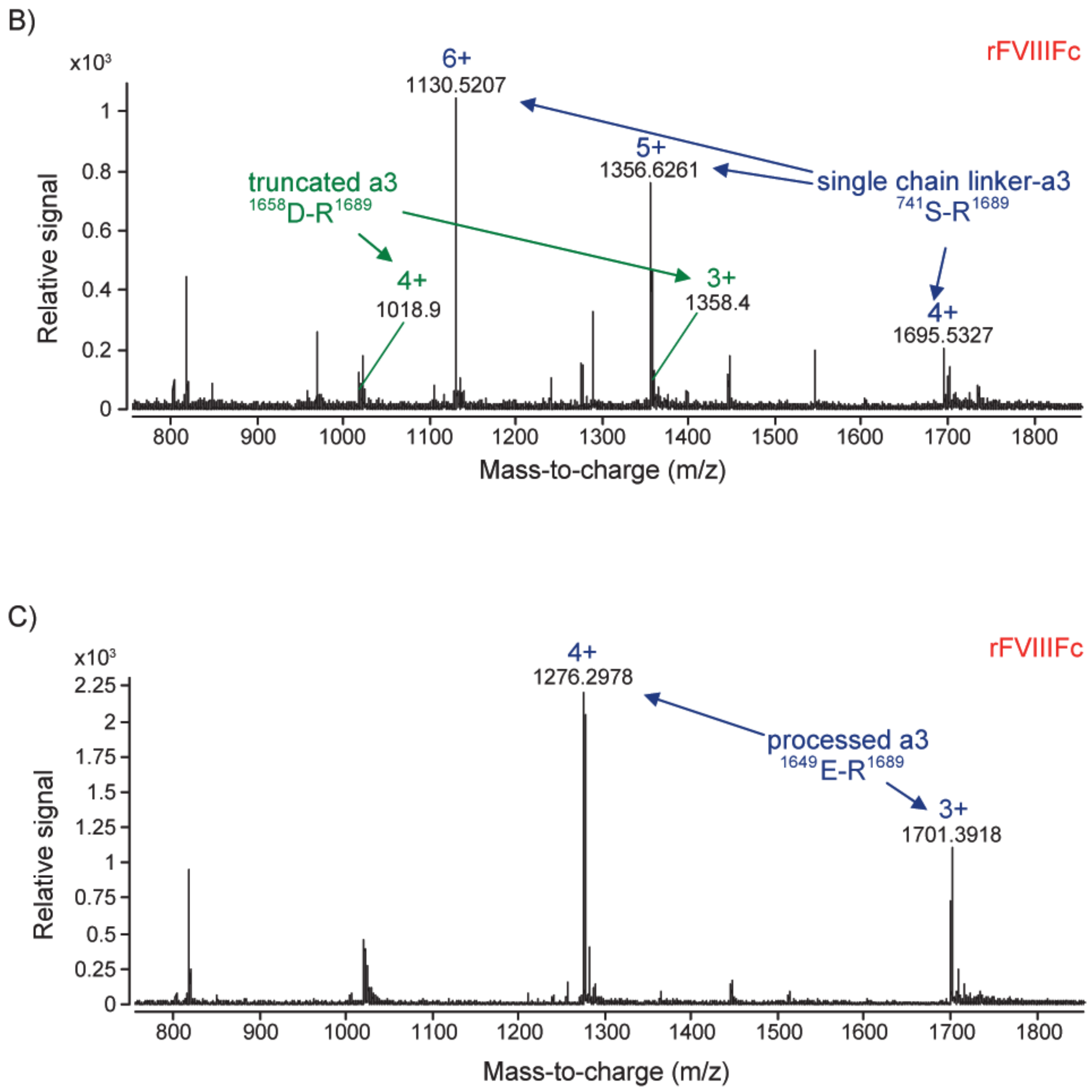
B)
rFVIIIFc
x10³
Relative signal
Mass-to-charge (m/z)
6+
1130.5207
5+
1356.6261
single chain linker-a3
741S-R1689
4+
1695.5327
truncated a3
1658D-R1689
4+
1018.9
3+
1358.4
C)
rFVIIIFc
x10³
Relative signal
Mass-to-charge (m/z)
4+
1276.2978
processed a3
1649E-R1689
3+
1701.3918

D)

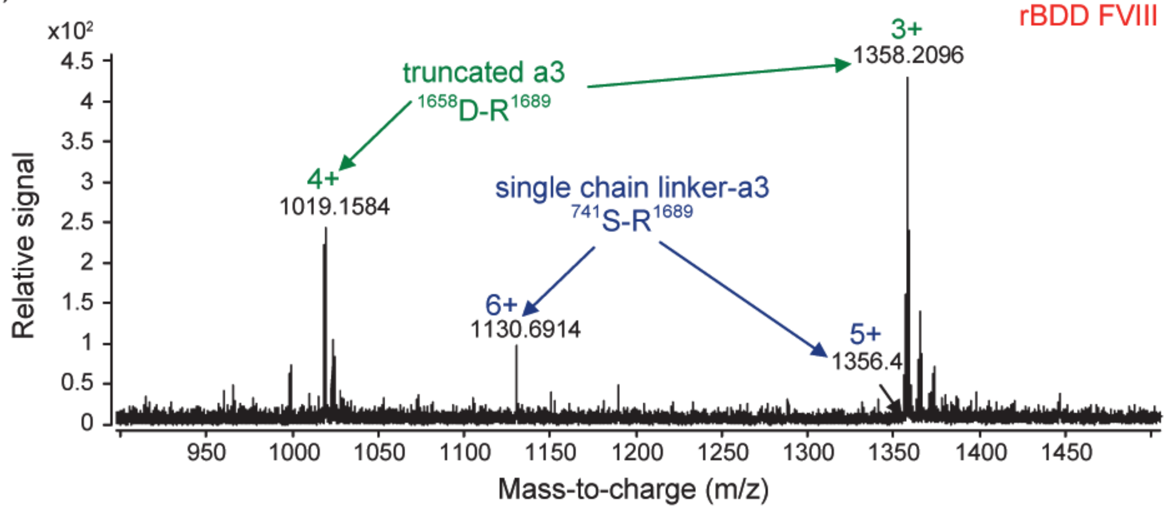
rBDD FVIII
x10²
truncated a3
1658D-R1689
4+
1019.1584
3+
1358.2096
single chain linker-a3
741S-R1689
6+
1130.6914
5+
1356.4
Relative signal
Mass-to-charge (m/z)

E)

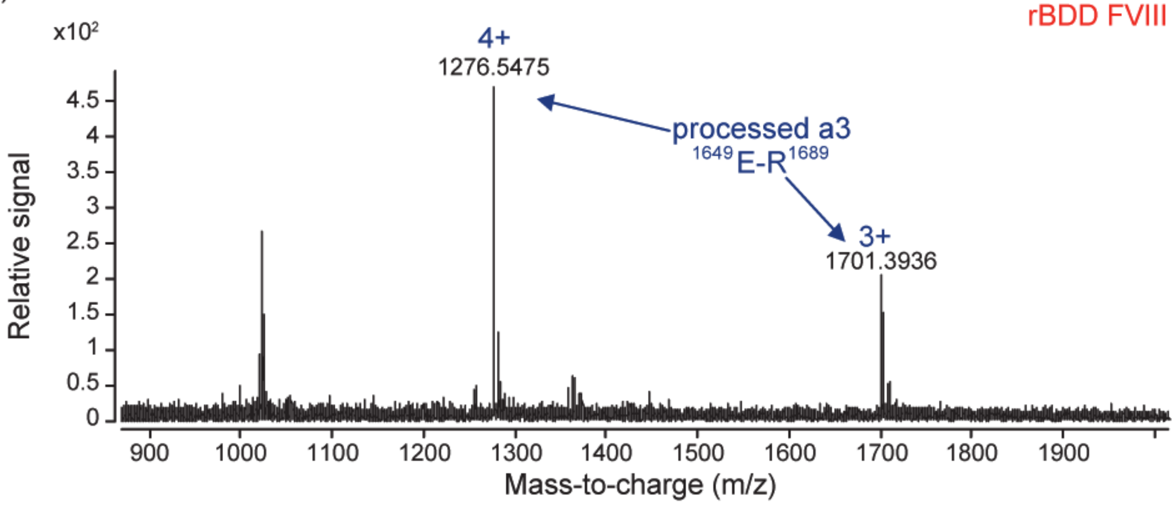
rBDD FVIII
x10²
4+
1276.5475
processed a3
1649E-R1689
3+
1701.3936
Relative signal
Mass-to-charge (m/z)

Fig. S3. Surface plasmon resonance sensorgrams that depict the interactions of (A) rFVIIIFc and (B) rBDD FVIII with immobilized hVWF. Double reference subtracted data (black lines) are overlaid with the best fit to a 1:1 model of association (red lines). FC, flow cell.

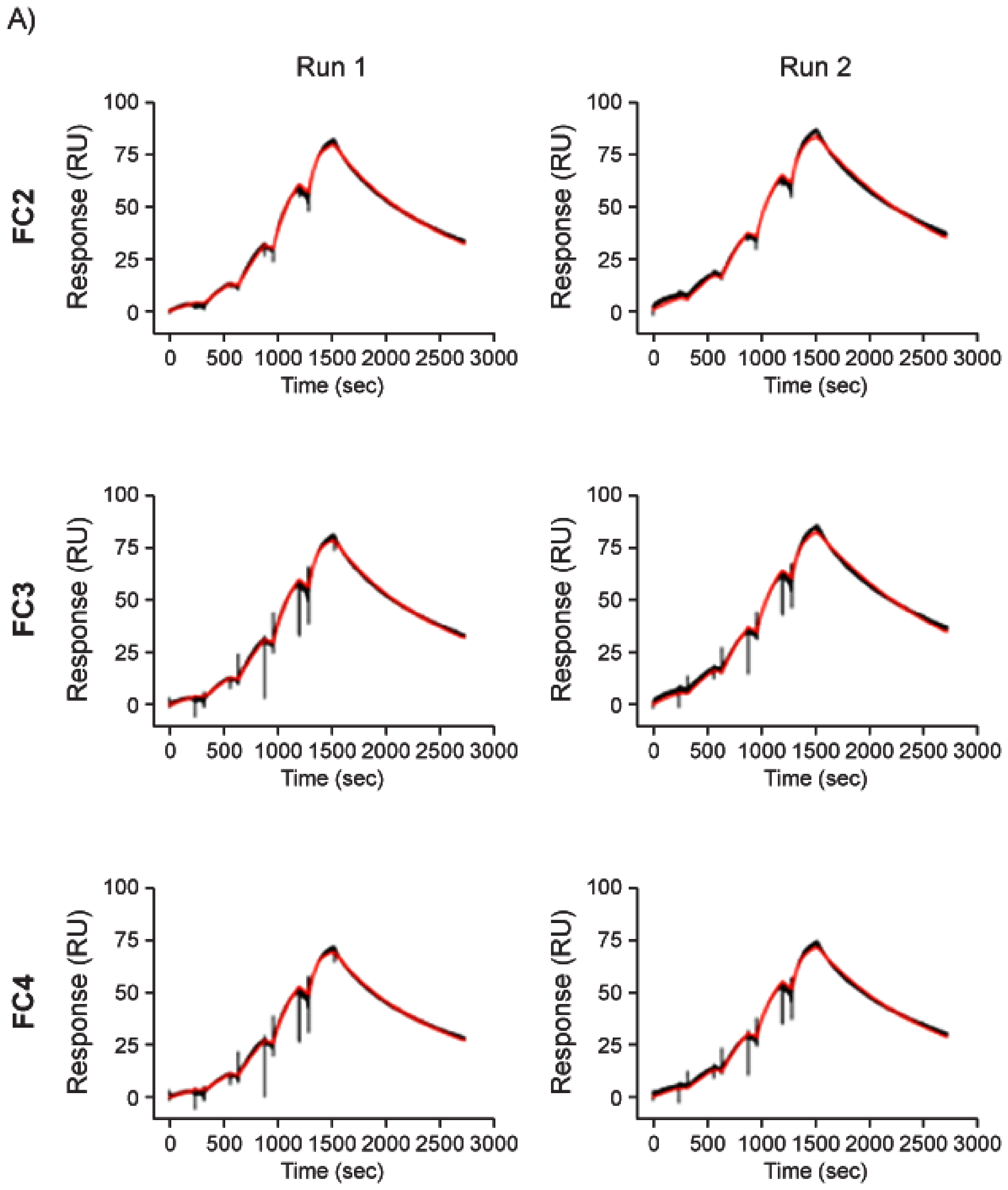

B)

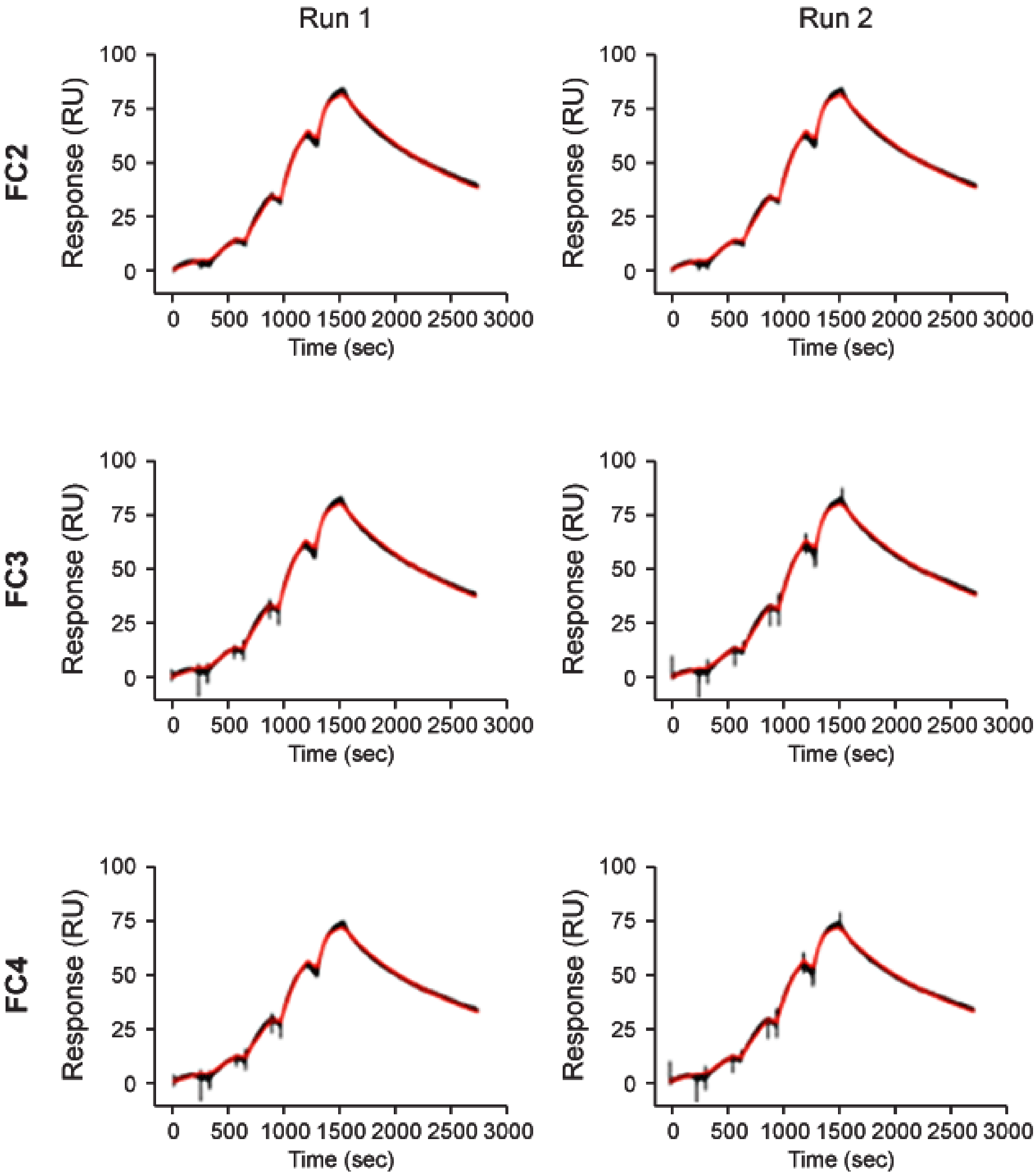
Run 1
Run 2
FC2
FC3
FC4
Response (RU)
Time (sec)
0
25
50
75
100
0
500
1000
1500
2000
2500
3000

References

1 McCue JT, Selvitelli K, Walker J. Application of a novel affinity adsorbent for the capture and purification of recombinant factor VIII compounds. *J Chromatogr A*. 2009; **1216**: 7824-30.

2 Chang JY, Monroe DM, Stafford DW, Brinkhous KM, Roberts HR. Replacing the first epidermal growth factor-like domain of factor IX with that of factor VII enhances activity in vitro and in canine hemophilia B. *J Clin Invest*. 1997; **100**: 886-92.

3 Karlsson R, Katsamba PS, Nordin H, Pol E, Myszka DG. Analyzing a kinetic titration series using affinity biosensors. Anal Biochem. 2006; **349**:136-47.
